# Use of Digital Health Interventions for Cancer Prevention Among People Living With Disabilities in the United States: A Scoping Review

**DOI:** 10.1002/cam4.70571

**Published:** 2025-01-16

**Authors:** Chinenye Lynette Ejezie, Lea Sacca, Sylvia Ayieko, Sara Burgoa, Yasmine Zerrouki, Diana Lobaina, Goodness Okwaraji, Christine Markham

**Affiliations:** ^1^ Department of Health Sciences Towson University Towson Maryland USA; ^2^ Charles E. Schmidt College of Medicine, Florida Atlantic University Boca Raton Florida USA; ^3^ University of Texas Health Science Center at Houston School of Public Health Houston Texas USA

**Keywords:** cancer prevention, digital health, people with disabilities, scoping review

## Abstract

**Background:**

The use of digital health strategies for cancer care increased dramatically in the United States over the past 4 years. However, a dearth of knowledge remains about the use of digital health for cancer prevention for some populations with heath disparities. Therefore, the purpose of the present scoping review was to identify digital health interventions for cancer prevention designed for people with disabilities.

**Methods:**

This scoping review was guided by the Preferred Reporting Items for Systematic Reviews and Meta‐Analyses extension for scoping reviews and the Arksey and O'Malley methodological framework. The Embase, PubMed, Ovid MEDLINE, and CINAHL/EBSCO databases were searched for peer‐reviewed articles published from database inception to February 5, 2024. Reports published in English of studies that employed digital health strategies for cancer prevention, were conducted among people with disabilities regardless of age, and were conducted in the United States were included.

**Findings:**

Following screening for eligibility, seven articles were identified. The types of disabilities were cancer (*n* = 4), bipolar I or II disorder (*n* = 1), obesity (*n* = 1), and deafness (*n* = 1). Interventions focused on education (*n* = 4), screening (*n* = 3), smoking cessation (*n* = 3), physical activity (*n* = 1), and cessation support (*n* = 1). Digital health strategies consisted of educational content delivered online, text messaging, interactive educational games, and downloadable informational applications. The common outcome of interest across all manuscripts was intervention efficacy.

**Interpretation:**

Overall, limited research is available to evaluate the use of digital health for cancer prevention among people with disabilities. This review identified gaps in knowledge that, if addressed, may help guide continued innovation in the use of digital health strategies for cancer prevention among people with disabilities.

## 1 Introduction

The application of digital health strategies in the design of cancer prevention interventions has become popular in the United States, with a dramatic increase over the past 4 years [1, 2, 3, 4]. Digital health strategies are either used alone or in combination with other methods, such as face‐to‐face hospital visits [5, 6]. Digital health can be described as “health services and information to manage illness and health risks delivered or enhanced through the internet and related technologies, that is, information and communication technology.” [7] Digital health encompasses telemedicine, telehealth, telemonitoring, mobile health, digital therapeutics, and digital health analytics [7, 8]. Digital health may improve quality of and access to health care and the efficiency of the healthcare sector [9]. It has also been associated with other advantages over face‐to‐face hospital visits, including accessibility, feasibility, scalability, and cost‐effectiveness [10].

Whereas researchers have studied the use of digital health for cancer prevention for populations with health disparities, including racial and ethnic minority groups [11], research is limited regarding the use of digital health for cancer prevention among people with disabilities, who were designated as a population with health disparities by the US National Institute on Minority Health and Health Disparities on September 26, 2023 [12]. In the United States, people with disabilities account for 27% of the total population [13]. According to the US Centers for Disease Control and Prevention, “disability is any condition of the body or mind (impairment) that makes it more difficult for the person with the condition to do certain activities (activity limitation) and interact with the world around them (participation restrictions).” [14] Disabilities can involve mental health, communication, hearing, mobility, vision, learning, and other aspects of how people interact with each other and their environment [14].

People with disabilities are often affected disproportionately by social determinants of diseases that influence their health as well as their access to health care [15]. As a result, people with disabilities may have challenges accessing cancer prevention interventions. Also, limited access to health care may result in delays in detection, diagnosis, and treatment for people with disabilities who suffer from cancer, which can lead to detrimental health outcomes. Given the convenience and feasibility of digital health combined with its effectiveness for other populations with health disparities, specifically, racial and ethnic minority groups and people of low socioeconomic status [16, 17, 18], it may also be useful for people with disabilities and thus may be helpful in overcoming barriers in accessing health care. For example, by providing people with health care in the comfort of their homes [19], digital health may allow people with disabilities to avoid barriers such as transportation difficulties, inaccessible physical environments, and lack of assistive technology [20, 21].

Some barriers may deter people with disabilities from using digital health [22]. For example, people with intellectual disabilities may have linguistic and cognitive limitations that impair their ability to use text‐rich applications [22]. Also, physical obstacles may make the use of devices such as a computer keyboard difficult for people with physical disabilities [22]. Nevertheless, prior research on the use of electronic devices demonstrated that people with mild to moderate disabilities can overcome hurdles associated with the use of electronic devices and can learn to use the basic aspects of electronic devices [22, 23, 24]. Also, a study on the use of telemedicine by people with disabilities in 2021 demonstrated that about 35% of people with hearing disabilities and 43% of those with mobility disabilities used this strategy [25].

Because the popularity of digital health in cancer prevention has been rapidly increasing in scope, we sought to broaden the horizon of research in this area. Reviews of digital health for cancer prevention conducted to address health disparities have focused on racial/ethnic minority groups [11, 26, 27, 28, 29, 30]. Therefore, in this scoping review, we aimed to identify the digital health interventions used for cancer prevention among people with disabilities. Additionally, we aimed to identify the digital health strategies that have been employed for cancer prevention among people with disabilities, and the outcomes utilized to assess intervention effectiveness.

## 2 Methods

This scoping review was conducted by health researchers with expertise in cancer prevention research and in the development, implementation, and evaluation of digital health cancer prevention interventions in the United States. Because the goal of this research was to compare the effectiveness of digital health interventions for cancer prevention among people with disabilities in the United States rather than diverse populations with differing characteristics across the globe, only studies conducted in the United States were included. Limiting the review to US‐based studies helps to minimize variations across countries, such as economic differences that could be introduced from cross‐comparison of global disability population groups. The protocol for conducting this scoping review was registered with the Open Science Framework (https://doi.org/10.17605/OSF.IO/EKH26) on April 19, 2024. An electronic literature search was conducted to identify peer‐reviewed research articles published from database inception to February 5, 2024. The approach to conducting and reporting this scoping review was guided by the Preferred Reporting Items for Systematic Reviews and Meta‐Analyses (PRISMA) extension for scoping reviews and the Arksey and O'Malley methodology for scoping reviews developed at the University of York [31, 32]. Their methodology comprises five steps: (1) identify research questions, (2) search for relevant studies, (3) select studies relevant to the research questions, (4) chart the data, and (5) collate, summarize, and report the results. Detailed information on the PRISMA for scoping reviews [31] and the Arksey and O'Malley methodology [32] can be found elsewhere.

### 2.1 Step 1. Identify Research Questions

The three guiding research questions for the scoping review were as follows: (1) What digital health interventions for cancer prevention exist for people with disabilities? (2) Which digital health strategies have been employed for cancer prevention among people with disabilities? (3) What are the main/common outcomes measured?

### 2.2 Step 2. Search for Relevant Studies

A systematic search strategy including keywords and MeSH terms was developed by the research team to identify relevant literature. To narrow, widen, and combine literature searches, educational subject headings were used. Four electronic databases (Embase, PubMed, Ovid MEDLINE, and CINAHL/EBSCO) selected for their focus on cancer prevention interventions were searched to identify peer‐reviewed literature from primary and secondary data sources, including case reports. Core search terms for digital health were combined with search terms for disability and cancer prevention. Search terms were combined using the Boolean operator “AND” (Table 1). Titles and abstracts were screened using the Rayyan platform [33], and the two reviewers (CLE and SA) were blinded to each other's decisions to avoid bias. The Rayyan platform helps researchers working on scoping reviews to simplify the process of screening and article selection. Decisions on which abstracts to include or exclude were made based on the set inclusion and exclusion criteria. After individualized screening was finalized, blinding was removed, and conflicts were resolved through discussion among reviewers and involving a third reviewer (LS) to reach consensus. The individualized screening was completed over a 2‐month period ending in February 2024. Conflicts were resolved over a period of 2 weeks ending in March 2024.

**TABLE 1:** Terms used to search for relevant studies.

Keywords	Search terms
Digital health intervention	Text message, mobile applications, Internet, digital intervention, web‐based intervention, e‐health intervention, digital, mobile, mHealth, telemedicine
Disability	People living with disability, people with disability, disability
Cancer prevention	Prevent, cancer

#### 2.2.1 Inclusion Criteria

Peer‐reviewed studies with findings published in English that (1) employed digital health interventions for cancer prevention, (2) were conducted among people with disabilities regardless of age, and (3) were conducted in the United States were included. All disabilities covered by the Americans with Disabilities Act (ADA) were considered [34]. Examples of these disabilities are bipolar disorder, cancer, deafness, and obesity (if it causes impairments).

#### 2.2.2 Exclusion Criteria

Studies that included people without disabilities, were conducted outside the United States, and described interventions for which the use of digital health was not clearly reported were excluded. Additionally, manuscripts focused on technologies used in collecting health information (e.g., remote patient monitoring) without a clear indication of cancer prevention were excluded.

### 2.3 Step 3: Select Studies Relevant to the Research Questions

**FIGURE 1:**
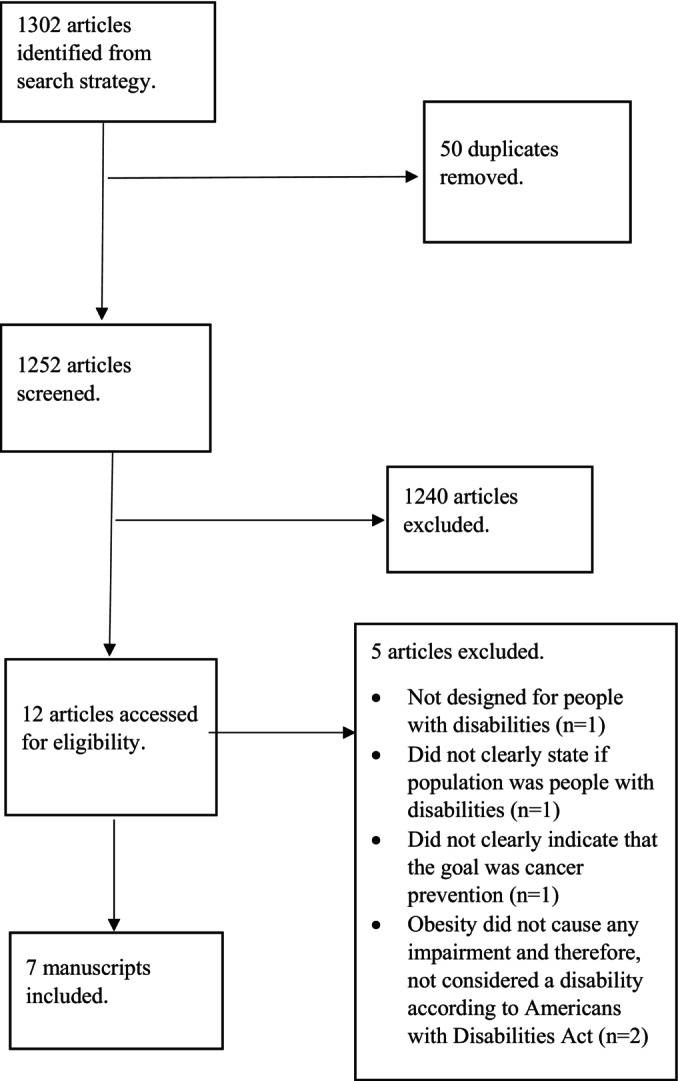
PRISMA extension for scoping reviews diagram.

The PRISMA diagram shown in Figure 1 depicts the process of selecting manuscripts for this review. The initial search identified 1302 articles. Of those articles, 1159 were found in PubMed, 16 were found in EBSCOhost, 69 were found in Ovid MEDLINE, and 58 were found in Embase. A total of 50 duplicate manuscripts were removed, leaving 1252 articles. The abstracts and titles of the 1252 articles were screened, and 1240 manuscripts were excluded for not meeting the inclusion criteria, leaving 12 manuscripts for full‐text review. Two of the authors (C.L.E. and S.A.) conducted the full‐text review, discussed disagreements about the articles, and arrived at a consensus. Following the full‐text review, five articles were excluded for not meeting the inclusion criteria (see Figure 1).

### 2.4 Steps 4 and 5: Chart the Data and Collate, Summarize, and Report the Results

Data were extracted independently by four authors (S.B., Y.Z., D.L., G.O.), with discrepancies resolved by C.L.E. or L.S. The following study characteristics were tabulated: primary author, study design, sample size, study population, study purpose, type of disability, type of cancer, and main prevention efforts (Table 2). The research methods and analytical strategies, digital health strategies employed for cancer prevention, and major intervention outcomes are listed in Table 3.

**TABLE 2:** Study characteristics.

Article no.	Reference	Study design	Sample size (*n*)	Study population	Study purpose	Theoretical framework or construct	Type of disability	Main prevention efforts
1	Heffner et al. [35]	Randomized Controlled Trial	51	Daily smokers with bipolar I or II disorder at four US sites (in Colorado, California, and Massachusetts)	To evaluate a novel, targeted, web‐based intervention for smokers with bipolar disorder based on ACT that was designed for reach and disseminability versus smokefree.gov	Acceptance and Commitment Therapy Theory	Bipolar I or II disorder	Smoking cessation
2	Krebs et al. [36]	Randomized Controlled Trial	42	Memorial Sloan‐Kettering Cancer Center patients who smoked cigarettes within the last 30 days and were scheduled for surgical treatment	To examine the feasibility (recruitment and retention rates), acceptability (patient satisfaction), quitting self‐confidence, and other cessation‐related indices of a cessation app targeting tobacco‐dependent cancer patients. The app included game design and behavioral rehearsal principles to help smokers identify, model, and practice coping strategies to avoid relapse of smoking	Social Cognitive Theory	Cancer (lung or gastrointestinal cancer, among others)	Smoking cessation
3	Leone et al. [37]	Randomized Controlled Trial	181	Nonobese (BMI, 18·5–29·9 kg/m^2^) and obese (BMI, 30+ kg/m^2^) White women aged 50 years and older	To determine whether, compared with nontailored (generic) messages, targeted messages are more relevant, acceptable, and more likely to improve colorectal cancer screening and physical activity behaviors among women with obesity	Knowledge, perceived susceptibility, perceived barriers, perceived benefits, and self‐efficacy	Obesity	Education, Screening and Physical Activity
4	Manne et al. [38]	Randomized Controlled Trial	441	Individuals recruited from Rutgers Cancer Institute of New Jersey, dermatology practices associated with Rutgers Robert Wood Johnson Medical School, Saint Barnabas Medical Center, and the New Jersey State Cancer Registry diagnosed with stage 0‐III melanoma who underwent surgery 3–24 months prior to the intervention, had not done thorough SSEs in the past 2 months, and/or did not adhere to sun protection recommendation	To evaluate the impact of the online intervention MSS on engagement in SSE and sun protection behaviors among melanoma survivors	Knowledge, self‐efficacy, benefits, barriers, and controllability	Cancer (melanoma)	Education and Screening (SSE and sun protection)
5	Palmer et al. [39]	Randomized controlled trial	150	Deaf ASL‐users recruited nationally in the United States via deaf clubs, organizations, community events, and a previous genetic counseling and testing study	To compare the effectiveness of online cancer genetics information presented using a bilingual approach (ASL with English closed captioning) versus a monolingual approach (English text)	Not specified	Deafness	Education
6	Rettig et al. [40]	Randomized controlled trial	29	Smokers diagnosed with head and neck or thoracic cancers undergoing radiation therapy at Johns Hopkins cancer treatment centers	To evaluate the efficacy of a novel smoking cessation intervention designed for upper aerodigestive cancer patients undergoing radiation therapy	Not specified	Cancer (upper aerodigestive cancers, specifically, head and neck or thoracic cancers)	Smoking cessation; Treatment outcomes; Cessation support
7	Vogel et al. [41]	Randomized controlled trial	104	Women with epithelial ovarian, primary peritoneal or fallopian tube cancer who did not undergo or schedule genetic counseling or testing related to cancer previously	To assess the feasibility and effectiveness of the week‐long mAGIC intervention aimed at persuading women with ovarian cancer to pursue genetic counseling	Health Belief Model	Cancer (epithelial ovarian, primary peritoneal, or fallopian tube cancer)	Education, Screening

Abbreviations: ACT, acceptance and commitment therapy; BMI, body mass index.

**TABLE 3:** Digital health strategies and intervention outcomes.

Article no.	Reference	Type of methodology/Analysis used	Digital health strategies	Major outcomes of the intervention
1	Heffner et al. [35]	Descriptive statistics; generalized linear mixed effects models	Web‐based smoking cessation interventions (WebQuit Plus and Smokefree.gov) supplemented by nicotine patch treatment	The WebQuit Trial design showed favorable retention rates, however, alternative recruitment methods are needed for larger trials Intervention led to smoking cessation outcomes, with 12% abstinence at the end of treatment for WebQuit Plus versus 8% for Smokefree.gov The overall quit rates may have been negatively impacted by low efficacy of the pharmacotherapy Cessation does not negatively impact the mental health of smokers with bipolar disorder based on findings that (1) psychiatric symptoms either remained the same or improved slightly over time and that (2) psychiatric adverse events showed no clear relationship with the intervention
2	Krebs et al. [36]	Descriptive statistics	The QuitIT game, a digital app provided on an iPad device, designed to promote smoking cessation through interactive gameplay that simulates situations and coping strategies related to quitting smoking	There were recruitment and retention difficulties in the trial, suggesting challenges in delivering a smoking cessation app‐based intervention during the perihospitalization period Recruitment of cancer patients is complicated due to several factors such as patients’ anxiety, patients worrying about their treatment outcomes, and disruptions in daily routines owing to multiple medical appointments Description of the app as a “game” might have impacted rates of study participation as it was perceived as frivolous in the context of cancer care Satisfaction with the game was largely positive among those who played it Researchers observed a nonsignificant trend of increased confidence to quit in the intervention group. More people abstained in the intervention group than in the standard care arm at 1‐month of follow‐up Gameplay during hospitalization and recovery may be difficult owing to health‐related challenges such as healing process and physical symptoms that adversely affect quality of life
3	Leone et al. [37]	Logistic regression; moderation and subgroup analysis	Internet‐delivered messages, both those targeted to obese women and generic messages, to promote health behaviors	No significant differences in message elaboration, behavioral intentions, relevance, or trustworthiness between the intervention and control groups among either weight group were found Exercise intentions increased at a higher rate among inactive women with obesity who received intervention messages compared with those who did not receive the intervention Elaboration was higher among women with obesity than women without obesity regardless of the type of message they received Although not statistically significant, colorectal cancer screening intentions were higher among unscreened women with obesity in the intervention group Tailoring interventions for women with obesity may be more effective than a nontailored approach
4	Manne et al. [38]	Logistic/standard regression; mediation analysis	MSS is a behaviorally based program delivered via the Internet, tailored to the user, and fully automated with no human clinical support. The program included orientation, three core sections, and a body mole map for tracking skin growths	The online intervention MSS showed consistent and durable effects on SSE among melanoma survivors, and demonstrated high utilization among affected individuals MSS had a significant positive effect on thorough SSE but mixed results regarding sun protection behaviors Participants in the MSS condition were more likely to report conducting a thorough SSE across all follow‐up assessments than were those receiving usual care Regarding sun protection behaviors, significant improvements were observed in the MSS condition at 24 weeks in both multivariate and univariate analyses, but the effect at 48 weeks was significant only in multivariate analyses Knowledge, barriers, and self‐efficacy served as mechanisms for effects and provide support for the Preventive Health Model
5	Palmer et al. [39]	*T*‐tests; chi‐squared/Fisher exact tests; regression analysis; McNemar test; analysis of variance	Educational content delivered online, using videos in ASL with closed captioning for the bilingual modality and written English text for the monolingual modality	The bilingual approach significantly increased knowledge scores for participants with low education The bilingual modality resulted in greater confidence in creating a family tree. The modality also positively influenced intentions to adhere to doctor's recommendation to either see a genetic counselor or undergo cancer genetic testing. Additionally, the bilingual modality approach resulted in increased intention to encourage others to seek genetic counseling for cancer risk and increase in the rate at which health information was recommended to family and friends Lower educated deaf ASL‐users have a better chance of accessing cancer genetic information via a bilingual; approach than does a monolingual approach
6	Rettig et al. [40]	Fisher exact *t*‐test; time‐series panel regression analysis	Text messaging as a component of comprehensive smoking cessation support	The Intervention group had markedly higher smoking abstinence rates at 8 weeks than did the Control group. Participants in the intervention group were also more likely to maintain reduced smoking intensity during the first 8 weeks of the study Observed short‐term decrease in smoking may be attributable to the comprehensive nature of the intervention Effective smoking cessation interventions delivered concurrently with radiation therapy may improve survival outcomes Two modifiable risk factors for smoking identified during the study are history of depression and current pain
7	Vogel et al. [41]	Chi‐squared tests; Fisher exact tests; two‐sample, two‐sided *t*‐tests	The mAGIC mobile application, which was designed based on the Fogg Behavior Model and the Health Belief Model to provide education and increase use of genetic counseling services	Use of cancer genetic counseling services improved in both study arms over that among the historical controls, but the difference was not statistically significant Women receiving the intervention demonstrated greater levels of knowledge of hereditary cancer and higher willingness to talk with their family about genetic counseling than the control subjects. Women who received the intervention also indicated greater self‐efficacy in scheduling a genetic counseling appointment than those in the control group

Abbreviation: NRT = nicotine replacement therapy.

## 3 Results

We reviewed a total of seven studies [35, 36, 37, 38, 39, 40, 41]. Four of the studies were published from 2019 to 2024. All seven study designs were randomized control trials. The sample sizes ranged from 29 to 441 people with disabilities living in the United States. The types of disabilities included in the articles were cancer (*n* = 4), bipolar I or II disorder (*n* = 1), obesity (*n* = 1), and deafness (*n* = 1).

### 3.1 Digital Health Interventions for Cancer Prevention in People With Disabilities

The studies included were those of various digital health interventions for cancer prevention among people with disabilities (Table 2).

The digital health intervention identified for people with bipolar I or II disorder was the acceptance and commitment therapy–based smoking cessation program, WebQuit [35]. Digital health interventions for people with cancer included the QuitIT coping skills game, the Application for Genetic Information on Cancer (mAGIC), mySmartSkin (MSS), and smoking cessation interventions that included intensive counseling, education, pharmacotherapy, SMS messaging support, and financial incentives [36, 38, 40, 41]. Digital health interventions for people with deafness provided information through a bilingual approach using American Sign Language (ASL) with English closed captioning [39]. Finally, interventions designed for people with obesity consisted of Internet‐delivered targeted messages for colorectal cancer screening and physical activity promotion among obese women [37].

### 3.2 Digital Health Strategies Employed for Cancer Prevention Among People With Disabilities

The main prevention efforts employed to reduce cancer rates were education (*n* = 4), screening (*n* = 3), smoking cessation (*n* = 3), physical activity (*n* = 1), and cessation support (*n* = 1) (Table 2). The digital health strategies highlighted in the included studies comprised educational content delivered online, text messaging, interactive educational games, and downloadable informational applications (Table 3).

### 3.3 Main Intervention Outcomes Measured

Across the interventions presented in the articles, the authors noted several major outcomes and findings (Table 3). A consistent theme was the efficacy of tailored outreach and education within each digital intervention, as interventions tailored to the condition of the patient were more successful than those with less specific tailoring. For example, Palmer et al. [39] found that presenting cancer genetics information online using videos in ASL with closed captioning in bilingual modality significantly increased knowledge scores for participants with low education, whereas those in the monolingual modality did not significantly increase in their knowledge scores. Similarly, a study by Manne et al. [38] evaluating the impact of the online intervention, MSS, on engagement in skin self‐examination (SSE) and sun protection behaviors among melanoma survivors illustrated consistent, durable effects on self‐examination; high utilization and positive evaluation. MSS was tailored to each participant, further highlighting the importance of designing digital interventions for users. In contrast, Krebs et al. [36] found that QuitIT, their digital tobacco cessation app in the form of a game, was not properly tailored to particpants' medical situations, as using the app during perihospitalization and recovery periods may have been particularly challenging owing to the healing process and presence of physical symptoms adversely affecting energy and quality of life. Other studies, such as those by Leone et al. [37] and Heffner et al. [35], had positive results regarding the impact of digital health interventions on cancer prevention. However, recruitment issues and other barriers limited the ability to establish significant relationships. Overall, tailored approaches with digital health strategies promoted positive outcomes for people with disabilities. Using tailored, culturally conscious outreach methods along with leveraging technology showed promise in promoting various health screenings and interventions across the studies.

Of the studies reviewed, five were informed by theories or theoretical constructs. One employed Acceptance and Commitment Therapy Theory [35], one employed the Social Cognitive Theory [36], one employed the Health Belief Model [41], one employed the Fogg Behavior Model [41], and two employed constructs from the Health Belief Model [37, 38].

## 4 Discussion

To the best of our knowledge, this is the first scoping review of the use of digital health interventions for cancer prevention among people living with disabilities. The increase in digital health strategies for cancer prevention combined with the designation of people with disabilities as a population with health disparities prompted this review to identify gaps in the use of digital health strategies for cancer prevention among people living with disabilities. Our findings demonstrate that despite the surge in digital health strategies over the last 4 years, the use of these strategies for cancer prevention among people with disabilities remains minimal. This is exemplified by the fact that we identified only seven articles eligible for this scoping review. Research findings regarding digital health interventions for cancer prevention among other populations (e.g., people without disabilities, racial and ethnic minorities) may not be applicable for people with disabilities. Therefore, more disability‐related research is needed to understand how digital health strategies can be used to design cancer prevention interventions for people with disabilities. Analysis of these seven articles may help identify major gaps that, if addressed, could improve the use of digital health strategies for cancer prevention interventions among people living with disabilities.

### 4.1 Digital Health Interventions for Cancer Prevention in People With Disabilities

The included studies addressed the following disabilities: cancer, deafness, bipolar disorder, and obesity. However, several conditions that are considered disabilities according to the ADA were missing. For example, no study examined the use of digital health for cancer prevention among people with mobility disability, diabetes, human immunodeficiency virus, major depressive disorders, or autism spectrum disorder. This is a considerable gap given the high number of people with these disabilities in the United States. According to US disability estimates, about 12.1% of adults have a mobility disability [13], 11.6% of adults have diabetes [42], 1.2 million adults have human immunodeficiency virus [43], 18.4% of adults have depression [44], and 2.1% of adults have autism spectrum disorder [45]. The benefits of digital health strategies may help improve cancer prevention behaviors among people with these disabilities. Because people with disabilities were not included as a population of interest within the Healthy People Initiative until 2010 [46], digital health interventions designed prior to 2010 may have failed to stratify populations based on disability status, thus combining people with disabilities with those without disabilities. Also possible is that digital health interventions for cancer prevention focusing on people with these disabilities do not exist. Therefore, more research in this area is needed. Future research should identify cancer prevention interventions for people with these disabilities and determine how digital health strategies could be employed for them.

### 4.2 Digital Health Strategies Employed for Cancer Prevention Among People With Disabilities

Our findings on the use of digital health strategies demonstrated that only a few such interventions exist for people with disabilities. This may reflect that people with certain disabilities according to the ADA (e.g., blindness, intellectual disabilities) may have challenges using technology [22]. For example, people with intellectual disabilities have linguistic and cognitive limitations that may affect their ability to use technological programs such as text‐rich applications [22]. Also, people who are blind have visual limitations that may affect their ability to use digital devices [47, 48]. Thus, digital health strategies may not be feasible for them. Nevertheless, digital health strategies are useful for people with certain disabilities and may be helpful in improving cancer prevention behaviors. Also, given the rapid pace of technological development and the efforts technology experts are making to create digital devices useful for people with disabilities, including those who are blind [47, 48], continued performance of scoping reviews of the literature for the use of digital health strategies is imperative to stay current on technology advancements for people with disabilities and identify novel technologies that may be useful, particularly for cancer prevention among people with disabilities. Thus, more research is needed to improve the use of digital health for certain populations with disabilities.

Our findings on the types of cancer prevention efforts used in the reviewed studies demonstrate that education was the most common prevention method employed. This finding is unsurprising given that a high number of cancer prevention interventions include education as one of the strategies. In their research on cancer prevention strategies, Lopez and colleagues [49] concluded that the most successful cancer prevention interventions tend to combine education with other strategies.

All of the digital health interventions in this scoping review were created prior to 2020. This finding is troubling given the abrupt surge in the use of digital health for cancer care across populations over the past 4 years. It suggests that people with disabilities were left behind and that researchers focused on other populations (general population, racial and ethnic minorities, etc.) when designing cancer prevention interventions using digital health strategies. Thus, more research in this area is needed. Future research should focus on how digital health strategies could be employed in the design of cancer prevention interventions for people with disabilities.

In the seven studies reviewed, none of the researchers used emerging technologies such as wearable devices to design their digital health interventions. Given that some disability groups, including people with diabetes, have used wearable technology to manage their conditions [50], these technologies may also be useful for cancer prevention in this population. Also, authors have reported that the use of emerging technologies such as wearable devices for healthcare purposes is effective [51, 52, 53]. Therefore, this may be a useful strategy to employ when designing cancer prevention interventions for people with disabilities. Given the rapid pace at which technological devices are created to improve care for people with disabilities, public health experts must be up to date with these advancements to know which strategies could be used for cancer prevention interventions.

### 4.3 Main Intervention Outcomes Measured

In all the studies reviewed, efficacy or effectiveness of digital health interventions was an outcome of interest. The majority of the studies [35, 37, 38, 39, 40, 41] demonstrated that digital health interventions for cancer prevention are effective for people with disabilities. However, the studies had limitations that have implications for scalability, generalizability, and implementation. No article directly addressed the broad scalability, which is of particular interest because of the challenges people with disabilities may encounter when trying to gain access to digital devices. For example, Manne et al. [38] and Vogel et al. [41] mentioned that their participants were all non‐Hispanic White individuals and that their research was conducted in a small‐scale setting. Therefore, their intervention may not be scalable to people with disabilities who belong to racial and ethnic minority groups and/or to larger settings. Because non‐Hispanic White people form a historically advantaged racial and ethnic group, they are more likely to have access to better health care and technology than their racial and ethnic minority counterparts [54, 55]. Based on the fundamental cause theory, the health care and technology‐related inequities among racial and ethnic minority groups result from nonequivalence of social economic status indicators across racial categories [55, 56, 57, 58]. Specifically, racial and ethnic minority groups receive lower income at the same educational level when compared with their non‐Hispanic White counterparts and are thus more likely to report financial difficulties [55, 56, 57, 58]. Therefore, research on digital health interventions must be expanded among people with disabilities across all racial and ethnic minority groups. This will provide researchers with information pertaining to the relevance and effectiveness of their interventions across all racial and ethnic groups. Having information about scalability would allow researchers to know the types of challenges encountered when employing digital health strategies and how to address them. Therefore, more data on the scalability of digital health interventions for people with disabilities will be helpful to public health experts when considering, creating, and implementing such interventions.

Of the studies reviewed, researchers in four of them raised concerns about the generalizability of their findings. Heffner et al. [35] mentioned that their study had a small sample with low power and therefore may not be generalizable. Manne et al. [38] raised concerns about the generalizability of their findings owing to their participants primarily consisting of non‐Hispanic White people and therefore not being representative of the US population. Vogel and colleagues [41] noted that their study was conducted at a single academic center at which mostly women who are non‐Hispanic White, are educated, and have health insurance receive treatment and that their findings therefore may not be generalizable to other populations. Palmer et al. [39] mentioned that because their goal was to recruit as many people as possible, their findings may not necessarily generalize to those who do not have access to the Internet. The findings of these studies thus cannot be generalized to other populations across the United States. Therefore, more research of digital health for cancer prevention among people with disabilities using larger samples and wider demographics that better represent the population of the United States is needed.

Although researchers in one study mentioned that their intervention has the potential for future implementation in clinical and public health practice [38], no authors provided a detailed discussion of how their intervention can be implemented or their plans for implementation. Whereas digital health interventions may appear feasible during research, potential barriers can hinder their adoption in health care, such as limited resources, interoperability, and incongruency with existing workflows [59]. Thus, for researchers who employ these strategies, keeping implementation in mind from the onset of program development is important [59]. Approaches to program implementation adapted from implementation science framework could help to ensure that digital health interventions are successfully integrated into clinical and public health practice [59].

Across the digital health interventions in the studies we reviewed, a consistent theme was the importance of designing interventions tailored to a specific population. The finding that tailored digital health approaches promote positive outcomes mirrors previous research. For example, in their comparison of the effectiveness and cost efficiency of a tailored message intervention with those of a nontailored message intervention for increasing colorectal cancer screening among a nonadherent population, Hirai et al. [60] found that the tailored approach was more effective. Similarly, in their evaluation of the effectiveness of a tailored communication intervention to promote colonoscopy uptake among first‐degree relatives of colorectal cancer patients, Bai et al. [61] found that those who received the tailored intervention had markedly better uptake than did those who did not receive the intervention. Therefore, researchers should continue to develop digital health interventions for cancer prevention tailored to people with disabilities.

Of the seven studies in our review, only two [39, 40] had digital health programs designed without using theories or theoretical constructs. In these two studies, researchers may have used theories or theoretical constructs, but if they did, they did not report it explicitly. This improvement in the application of theories or theoretical constructs is similar to previous research, which revealed an increasing trend of designing digital interventions using theories or theoretical constructs [62]. Among the five studies in which researchers applied theories, constructs from the Health Belief Model were most commonly employed to guide the digital health intervention content development. This finding is similar to a previous finding suggesting that the Health Belief Model is commonly used to design digital health interventions for cancer prevention [62]. In one intervention in this scoping review, researchers found minimal merit of use of theories to guide interventions. Specifically, Krebs et al. [36] found that despite their application of the social‐cognitive theory, they observed a nonsignificant trend of increased confidence among participants in the intervention group regarding quitting tobacco use. Although this study may not have been effective despite being theory‐informed, the authors noted that describing the intervention as a “game” was frivolous and thus may have been a factor in the intervention's ineffectiveness. In the other studies in which the researchers employed theories [35, 37, 38, 41], the interventions produced positive changes, which is in line with prior research suggesting that theory‐based intervention helps increase the effectiveness of programs [63]. Theories and theoretical constructs provide information regarding the efficacy or effectiveness of an intervention [63, 64]. Therefore, researchers should endeavor to use them to guide the design of their digital health programs. Also, authors who employ theories or theoretical constructs should explicitly state which ones they use to guide the design of their programs.

### 4.4 Strengths and Limitations

This scoping review is unique because it is among the first to acknowledge people with disabilities as a population with health disparities. Also, it is the first scoping review of the use of digital health for cancer prevention among people with disabilities.

Our scoping review has some limitations. For example, some disability‐related search terms, such as “disability” and “people with disabilities,” were somewhat broad. Thus, we may have omitted interventions designed for specific groups, such as people who have diabetes, people living with human immunodeficiency virus, and people who have mobility disabilities, all of which are disability groups according to the ADA. Also, because English was the only common language among the authors, we excluded articles not written in English, and thus, we may have missed interventions reported in manuscripts written in other languages. Although we performed a comprehensive search of the most relevant databases, we did not include gray literature, trace the references in the included manuscripts, or perform hand searches of health journals. We addressed these limitations by including as many disability, cancer, and digital health key words as possible; using the PRISMA for scoping reviews guidelines; and extracting data independently. In future scoping reviews, authors should endeavor to expand upon their search terms to capture all conditions considered disabilities. We did not include information pertaining to the racial and ethnic composition of participants. Future research is needed to expand on this by looking at the existing digital health interventions for people with disabilities according to race and ethnicity. Additionally, because our study focused on interventions in the United States alone, the findings cannot be generalized to different population groups across the globe. Future research should expand on this review by examining digital health interventions for cancer prevention in other countries. Future reviews should also assess the effectiveness of interventions that take into account the variations across countries such as economic differences that could influence the use of digital health strategies for cancer prevention among people with disabilities.

## 5 Conclusions

The use of digital health interventions for cancer prevention has increased dramatically over the past 4 years; yet, researchers have not frequently employed this strategy for people with disabilities. The findings of this scoping review help identify subgroups of people with disabilities for whom digital health strategies are not being used for cancer prevention. Our findings also pinpoint areas where improvements can be made in terms of the types of digital health technology used. More research is warranted to determine how to use emerging technologies to design theory‐based digital health interventions tailored for cancer prevention among people with disabilities.

## Author Contributions

Chinenye Lynette Ejezie was the primary reviewer who conceptualized the study, carried out the preliminary search, extracted the data, conducted analysis, developed the draft for the manuscript, and critically reviewed the manuscript. Lea Sacca was a secondary reviewer who helped with tabulation and writing of the result, conducted analysis, and critically reviewed the manuscript. Christine Markham was also a secondary reviewer who critically reviewed the manuscript. Sylvia Ayieko helped with data collection and analysis. Sara Burgoa, Yasmine Zerrouki, Diana Lobaina, and Goodness Okwaraji helped with tabulation of information.

## Consent

All authors agreed to the publication of this manuscript.

## Conflicts of Interest

The authors declare no conflicts of interest.

## Data Availability

The data used in this scoping review are existing literature and publicly available.

## References

[1] P. E. Lonergan , S. L. Washington Iii , L. Branagan , et al., “Rapid Utilization of Telehealth in a Comprehensive Cancer Center as a Response to COVID‐19: Cross‐Sectional Analysis,” Journal of Medical Internet Research 22, no. 7 (2020): e19322. 32568721 10.2196/19322 PMC7340164

[2] K. M. Shaffer , K. L. Turner , C. Siwik , et al., “Digital Health and Telehealth in Cancer Care: A Scoping Review of Reviews,” Lancet Digital Health 5, no. 5 (2023): e316–e327. 37100545 10.1016/S2589-7500(23)00049-3 PMC10124999

[3] V. A. Marks , W. R. Hsiang , W. Umer , et al., “Access to Telehealth Services for Colorectal Cancer Patients in the United States During the COVID‐19 Pandemic,” American Journal of Surgery 224, no. 5 (2022): 1267–1273. 35701240 10.1016/j.amjsurg.2022.06.005 PMC9176198

[4] J. Yu , C. Petersen , S. Reid , S. T. Rosenbloom , and J. L. Warner , “Telehealth and Technology: New Directions in Cancer Care,” Cancer Journal 30, no. 1 (2024): 40–45. 38265926 10.1097/PPO.0000000000000692

[5] S. A. Brown , S. Patel , D. Rayan , et al., “A Virtual‐Hybrid Approach to Launching a Cardio‐Oncology Clinic During a Pandemic,” Cardio‐Oncology 7 (2021): 1–5. 10.1186/s40959-020-00088-2 PMC7803880 33441188

[6] V. R. Ramnath , L. Hill , J. Schultz , et al., “An In‐Person and Telemedicine “Hybrid” System to Improve Cross‐Border Critical Care in COVID‐19,” Annals of Global Health 87, no. 1 (2021), 10.5334/aogh.3108. PMC7792461 33505860

[7] K. Lee , S. Kim , S. H. Kim , et al., “Digital Health Interventions for Adult Patients With Cancer Evaluated in Randomized Controlled Trials: Scoping Review,” Journal of Medical Internet Research 6, no. 25 (2023): e38333. 10.2196/38333 PMC9862347 36607712

[8] M. Aapro , P. Bossi , A. Dasari , et al., “Digital Health for Optimal Supportive Care in Oncology: Benefits, Limits, and Future Perspectives,” Kompass Nutrition & Dietetics 1, no. 3 (2021): 72–90. 10.1007/s00520-020-05539-1 PMC7447627 32533435

[9] C. J. Butcher and W. Hussain , “Digital Healthcare: The Future,” Future Healthcare Journal 9, no. 2 (2022): 113–117. 10.7861/fhj.2022-0046 PMC9345235 35928188

[10] R. F. Muñoz , “Harnessing Psychology and Technology to Contribute to Making Health Care a Universal Human Right,” Cognitive and Behavioral Practice 29 (2022): 4–14.

[11] C. L. Ejezie , J. Choi , S. Ayieko , et al., “Digital Health Interventions for Cancer Prevention Among Racial and Ethnic Minority Groups in the United States: A Scoping Review,” Journal of Racial and Ethnic Health Disparities 8 (2024): 1–7. 10.1007/s40615-024-01958-6 38587751

[12] National Institute on Minority Health and Health Disparities , “People with Disabilities Designated as HD Population,” accessed February 29, 2024, https://nimhd.nih.gov/about/directors‐corner/messages/health‐disparities‐population‐designation.html.

[13] Centers for Disease Control and Prevention , “Disability Impacts All of Us Infographic,” accessed February 29, 2024, https://www.cdc.gov/ncbddd/disabilityandhealth/infographic‐disability‐impacts‐all.html.

[14] Centers for Disease Control and Prevention , “Disability and Health Overview,” accessed February 29, 2024, https://www.cdc.gov/ncbddd/disabilityandhealth/disability.html.

[15] C. Reeves and D. Collingridge , “Improving Cancer Care for People With Disabilities,” Lancet Oncology 23, no. 4 (2022): 446–447. 35358448 10.1016/S1470-2045(22)00147-4

[16] N. S. Gregory , A. P. Shukla , J. J. Noel , et al., “The Feasibility, Acceptability, and Usability of Telehealth Visits,” Frontiers in Medicine 10 (2023): 10. 10.3389/fmed.2023.1198096 PMC10394377 37538312

[17] C. Campos‐Castillo and D. Anthony , “Racial and Ethnic Differences in Self‐Reported Telehealth Use During the COVID‐19 Pandemic: A Secondary Analysis of a US Survey of Internet Users From Late March,” Journal of the American Medical Informatics Association 28, no. 1 (2021): 119–125. 32894772 10.1093/jamia/ocaa221 PMC7499625

[18] J. J. Flores Garcia , M. W. Reid , and J. Raymond , “Feasibility of Shared Telemedicine Appointments for Low SES Adolescents and Young Adults With T1D,” Diabetes 67, no. Supplement_1 (2018): 1325‐P.

[19] K. Denecke , R. May , E. M. Borycki , and A. W. Kushniruk , “Digital Health as an Enabler for Hospital@ Home: A Rising Trend or Just a Vision?,” Frontiers in Public Health 17, no. 11 (2023): 1137798. 10.3389/fpubh.2023.1137798 PMC9981936 36875371

[20] Centers for Disease Control and Prevention , “Disability and Health Disability Barriers,” accessed February 29, 2024, https://www.cdc.gov/ncbddd/disabilityandhealth/disability‐barriers.html.

[21] J. L. Bezyak , S. Sabella , J. Hammel , K. McDonald , R. A. Jones , and D. Barton , “Community Participation and Public Transportation Barriers Experienced by People With Disabilities,” Disability and Rehabilitation 42, no. 23 (2020): 3275–3283. 30991852 10.1080/09638288.2019.1590469

[22] K. Krysta , M. Romańczyk , A. Diefenbacher , and M. Krzystanek , “Telemedicine Treatment and Care for Patients With Intellectual Disability,” International Journal of Environmental Research and Public Health 18, no. 4 (2021): 1746. 33670152 10.3390/ijerph18041746 PMC7916831

[23] C. W. Li‐Tsang , M. Y. Lee , S. S. Yeung , A. M. Siu , and C. S. Lam , “A 6‐Month Follow‐Up of the Effects of an Information and Communication Technology (ICT) Training Programme on People With Intellectual Disabilities,” Research in Developmental Disabilities 28, no. 6 (2007): 559–566. 16979318 10.1016/j.ridd.2006.06.007

[24] D. K. Davies , S. E. Stock , and M. L. Wehmeyer , “Computer‐Mediated, Self‐Directed Computer Training and Skill Assessment for Individuals With Mental Retardation,” Journal of Developmental and Physical Disabilities 16 (2004): 95–105.

[25] S. J. Miller , J. R. Sly , K. B. Gaffney , Z. Jiang , B. Henry , and L. Jandorf , “Development of a Tablet App Designed to Improve African Americans' Screening Colonoscopy Rates,” Translational Behavioral Medicine 10, no. 2 (2020): 375–383. 30799495 10.1093/tbm/ibz014 PMC7237545

[26] C. Friedman and L. VanPuymbrouck , “Telehealth Use by Persons With Disabilities During the COVID‐19 Pandemic,” International Journal of Telerehabilitation 13, no. 2 (2021): e6402. 35646237 10.5195/ijt.2021.6402 PMC9098125

[27] B. N. Robinson , A. F. Newman , E. Tefera , et al., “Video Intervention Increases Participation of Black Breast Cancer Patients in Therapeutic Trials,” Npj Breast Cancer 18, no. 3 (2017): 36, 10.1038/s41523-017-0039-1. PMC5603544 28944289

[28] J. H. Wang , M. D. Schwartz , R. L. Brown , et al., “Results of a Randomized Controlled Trial Testing the Efficacy of a Culturally Targeted and a Generic Video on Mammography Screening Among Chinese‐American Immigrants,” Cancer Epidemiology, Biomarkers & Prevention 21, no. 11 (2012): 1923–1932, 10.1158/1055-9965.EPI-12-0821. PMC3542829 22971901

[29] A. C. Chen , S. W. Kim , L. Ou , M. Todd , and L. Larkey , “Digital Storytelling Intervention to Promote HPV Vaccination Among At‐Risk Asian Immigrant Populations: Pilot Intervention Study,” Journal of Medical Internet Research 4, no. 7 (2023): e46951, 10.2196/46951. PMC10585432 36877658

[30] W. Chee , Y. Lee , E. O. Im , et al., “A Culturally Tailored Internet Cancer Support Group for Asian American Breast Cancer Survivors: A Randomized Controlled Pilot Intervention Study,” Journal of Telemedicine and Telecare 23, no. 6 (2017): 618–626, 10.1177/1357633X16658369. 27486198 PMC6186171

[31] A. C. Tricco , E. Lillie , W. Zarin , et al., “PRISMA Extension for Scoping Reviews (PRISMA‐ScR): Checklist and Explanation,” Annals of Internal Medicine 169, no. 7 (2018): 467–473, 10.7326/M18-0850. 30178033

[32] H. Arksey and L. O'Malley , “Scoping Studies: Towards a Methodological Framework,” International Journal of Social Research Methodology 8, no. 1 (2005): 19–32.

[33] M. Ouzzani , H. Hammady , Z. Fedorowicz , and A. Elmagarmid , “Rayyan—A Web and Mobile App for Systematic Reviews,” Systematic Reviews 5, no. 1 (2016): 210, 10.1186/s13643-016-0384-4. 27919275 PMC5139140

[34] Americans with Disabilities Act , “Introduction to the Americans with Disabilities Act,” accessed March 19, 2024, https://www.ada.gov/topics/intro‐to‐ada/.

[35] J. L. Heffner , M. M. Kelly , J. Waxmonsky , et al., “Pilot Randomized Controlled Trial of Web‐Delivered Acceptance and Commitment Therapy Versus Smokefree. Gov for Smokers With Bipolar Disorder,” Nicotine and Tobacco Research 22, no. 9 (2020): 1543–1552. 31883336 10.1093/ntr/ntz242 PMC7443589

[36] P. Krebs , J. Burkhalter , J. Fiske , et al., “The QuitIT Coping Skills Game for Promoting Tobacco Cessation Among Smokers Diagnosed With Cancer: Pilot Randomized Controlled Trial,” JMIR mHealth and uHealth 7, no. 1 (2019): e10071. 30632971 10.2196/10071 PMC6329892

[37] L. A. Leone , M. K. Campbell , M. Allicock , and M. Pignone , “Colorectal Cancer Screening and Physical Activity Promotion Among Obese Women: An Online Evaluation of Targeted Messages,” Journal of Health Communication 17, no. 10 (2012): 1187–1203. 22775294 10.1080/10810730.2012.665422 PMC4201496

[38] S. L. Manne , C. J. Heckman , D. A. Kashy , et al., “Randomized Controlled Trial of the mySmartSkin Web‐Based Intervention to Promote Skin Self‐Examination and Sun Protection Among Individuals Diagnosed With Melanoma,” Translational Behavioral Medicine 11, no. 7 (2021): 1461–1472. 33904921 10.1093/tbm/ibaa103 PMC8320885

[39] C. G. Palmer , P. Boudreault , B. A. Berman , et al., “Bilingual Approach to Online Cancer Genetics Education for Deaf American Sign Language Users Produces Greater Knowledge and Confidence Than English Text Only: A Randomized Study,” Disability and Health Journal 10, no. 1 (2017): 23–32. 27594054 10.1016/j.dhjo.2016.07.002 PMC5136526

[40] E. M. Rettig , C. Fakhry , R. K. Hales , et al., “Pilot Randomized Controlled Trial of a Comprehensive Smoking Cessation Intervention for Patients With Upper Aerodigestive Cancer Undergoing Radiotherapy,” Head & Neck 40, no. 7 (2018): 1534–1547. 29542262 10.1002/hed.25148 PMC6037556

[41] R. I. Vogel , K. Niendorf , S. Petzel , et al., “A Patient‐Centered Mobile Health Application to Motivate Use of Genetic Counseling Among Women With Ovarian Cancer: A Pilot Randomized Controlled Trial,” Gynecologic Oncology 153, no. 1 (2019): 100–107. 30718125 10.1016/j.ygyno.2019.01.019 PMC12042298

[42] Centers for Disease Control and Prevention , “By the Numbers: Diabetes in America,” accessed April 20, 2024, https://www.cdc.gov/diabetes/health‐equity/diabetes‐by‐the‐numbers.html.

[43] Centers for Disease Control and Prevention , “Basic Statistics, HIV Basics, HIV/AIDS,” https://www.cdc.gov/hiv/basics/statistics.html.

[44] B. Lee , “National, State‐Level, and County‐Level Prevalence Estimates of Adults Aged≥ 18 Years Self‐Reporting a Lifetime Diagnosis of Depression—United States, 2020,” Morbidity and Mortality Weekly Report 72 (2023): 72–650. 10.15585/mmwr.mm7224a1 PMC10328468 37318995

[45] Centers for Disease Control and Prevention , “CDC Releases First Estimates of the Number of Adults Living with Autism Spectrum Disorder in the United States,” accessed April 20, 2024, https://www.cdc.gov/ncbddd/autism/features/adults‐living‐with‐autism‐spectrum‐disorder.html.

[46] K. Froehlich‐Grobe , L. Koon , C. Ochoa , and J. P. Hall , “Piloting the Effectiveness of the Workout on Wheels Internet Intervention (WOWii) Program Among Individuals With Mobility Disabilities,” Disability and Health Journal 24 (2024): 101636. 10.1016/j.dhjo.2024.101636 38670867

[47] B. Leporini , M. Rosellini , and N. Forgione , “Designing Assistive Technology for Getting More Independence for Blind People When Performing Everyday Tasks: An Auditory‐Based Tool as a Case Study,” Journal of Ambient Intelligence and Humanized Computing 11 (2020): 6107–6123.

[48] A. Khan and S. Khusro , “An Insight Into Smartphone‐Based Assistive Solutions for Visually Impaired and Blind People: Issues, Challenges and Opportunities,” Universal Access in the Information Society 20, no. 2 (2021): 265–298.

[49] A. M. Lopez , L. Hudson , N. L. Vanderford , R. Vanderpool , J. Griggs , and M. Schonberg , “Epidemiology and Implementation of Cancer Prevention in Disparate Populations and Settings,” American Society of Clinical Oncology Educational Book 17, no. 39 (2019): 50–60. 10.1200/EDBK_238965 PMC6556209 31099623

[50] J. S. Sherwood , S. J. Russell , and M. S. Putman , “New and Emerging Technologies in Type 1 Diabetes,” Endocrinology and Metabolism Clinics 49, no. 4 (2020): 667–678. 33153673 10.1016/j.ecl.2020.07.006 PMC7556222

[51] D. A. de Queiroz , C. A. da Costa , E. A. de Queiroz , E. F. da Silveira , and R. R. da Rosa , “Internet of Things in Active Cancer Treatment: A Systematic Review,” Journal of Biomedical Informatics 1, no. 118 (2021): 103814. 10.1016/j.jbi.2021.103814 34015540

[52] D. A. de Queiroz , R. S. Passarello , V. V. de Moura Fé , et al., “A Wearable Chatbot‐Based Model for Monitoring Colorectal Cancer Patients in the Active Phase of Treatment,” Healthcare Analytics 1, no. 4 (2023): 100257.

[53] A. McCracken , J. Harrison , and J. Hill , “Self‐Guided Technology to Improve Health‐Related Behaviour and Quality of Life in People With Cancer,” British Journal of Community Nursing 26, no. 9 (2021): 434–437. 34473558 10.12968/bjcn.2021.26.9.434

[54] K. Kan , N. Heard‐Garris , A. Bendelow , et al., “Examining Access to Digital Technology by Race and Ethnicity and Child Health Status Among Chicago Families,” JAMA Network Open 5, no. 8 (2022): e2228992. 36018593 10.1001/jamanetworkopen.2022.28992 PMC9419010

[55] A. M. Burdette , N. S. Webb , T. D. Hill , and H. Jokinen‐Gordon , “Race‐Specific Trends in HPV Vaccinations and Provider Recommendations: Persistent Disparities or Social Progress?,” Public Health 1, no. 142 (2017): 167–176. 10.1016/j.puhe.2016.07.009 27592005

[56] A. N. Polonijo and R. M. Carpiano , “Social Inequalities in Adolescent Human Papillomavirus (HPV) Vaccination: A Test of Fundamental Cause Theory,” Social Science & Medicine 1, no. 82 (2013): 115–125. 10.1016/j.socscimed.2012.12.020 23337830

[57] B. G. Link and J. Phelan , “Social Conditions as Fundamental Causes of Disease,” Journal of Health and Social Behavior 1 (1995): 80–94. 7560851

[58] D. R. Williams , N. Priest , and N. B. Anderson , “Understanding Associations Among Race, Socioeconomic Status, and Health: Patterns and Prospects,” Health Psychology 35, no. 4 (2016): 407–411. 27018733 10.1037/hea0000242 PMC4817358

[59] National Cancer Institute , “Realizing the Potential of Digital Health Through Implementation Science,” accessed November 11, 2024, https://cancercontrol.cancer.gov/is/about/blog/realizing‐potential‐digital‐health‐through‐implementation‐science.

[60] K. Hirai , Y. Ishikawa , J. Fukuyoshi , et al., “Tailored Message Interventions Versus Typical Messages for Increasing Participation in Colorectal Cancer Screening Among a Non‐adherent Population: A Randomized Controlled Trial,” BMC Public Health 16 (2016): 1–8. 27220976 10.1186/s12889-016-3069-y PMC4877938

[61] Y. Bai , C. L. Wong , X. Peng , K. C. Choi , and W. K. So , “Effectiveness of a Tailored Communication Intervention on Colonoscopy Uptake for Firstdegree Relatives of Colorectal Cancer Patients: A Randomized Controlled Trial,” Asia‐Pacific Journal of Oncology Nursing 9, no. 9 (2022): 100068. 35651882 10.1016/j.apjon.2022.04.007 PMC9149019

[62] J. Choi , I. Tami‐Maury , P. Cuccaro , S. Kim , and C. Markham , “Digital Health Interventions to Improve Adolescent HPV Vaccination: A Systematic Review,” Vaccine 11, no. 2 (2023): 249. 10.3390/vaccines11020249 PMC9963303 36851127

[63] K. Glanz and D. B. Bishop , “The Role of Behavioral Science Theory in Development and Implementation of Public Health Interventions,” Annual Review of Public Health 31 (2010): 399–418, 10.1146/annurev.publhealth.012809.103604. 20070207

[64] M. Eccles , J. Grimshaw , A. Walker , M. Johnston , and N. Pitts , “Changing the Behavior of Healthcare Professionals: The Use of Theory in Promoting the Uptake of Research Findings,” Journal of Clinical Epidemiology 58, no. 2 (2005): 107–112, 10.1016/j.jclinepi.2004.09.002. 15680740

